# Impact of type of full-field digital image on mammographic density assessment and breast cancer risk estimation: a case-control study

**DOI:** 10.1186/s13058-016-0756-7

**Published:** 2016-09-26

**Authors:** Marta Cecilia Busana, Amanda Eng, Rachel Denholm, Mitch Dowsett, Sarah Vinnicombe, Steve Allen, Isabel dos-Santos-Silva

**Affiliations:** 1 Department of Non-Communicable Disease Epidemiology, London School of Hygiene and Tropical Medicine, Keppel Street, London, WC1E 7HT UK; 2 Centre for Public Health Research, Massey University, Wellington, New Zealand; 3 Academic Biochemistry, Royal Marsden Hospital, London, UK; 4 Cancer Research, Ninewells Hospital Medical School, University of Dundee, Dundee, UK; 5 Department of Imaging, Royal Marsden NHS Foundation Trust, London, UK

**Keywords:** Digital mammography, Mammographic density, Breast density, Breast cancer, Image acquisition

## Abstract

**Background:**

Full-field digital mammography, which is gradually being introduced in most clinical and screening settings, produces two types of images: raw and processed. However, the extent to which mammographic density measurements, and their ability to predict breast cancer risk, vary according to type of image is not fully known.

**Methods:**

We compared the performance of the semi-automated Cumulus method on digital raw, “analogue-like” raw and processed images, and the performance of a recently developed method - Laboratory for Breast Radiodensity Assessment (LIBRA) - on digital raw and processed images, in a case-control study (414 patients (cases) and 684 controls) by evaluating the extent to which their measurements were associated with breast cancer risk factors, and by comparing their ability to predict breast cancer risk.

**Results:**

Valid Cumulus and LIBRA measurements were obtained from all available images, but the resulting distributions differed according to the method and type of image used. Both Cumulus and LIBRA percent density were inversely associated with age, body mass index (BMI), parity and postmenopausal status, regardless of type of image used. Cumulus percent density was strongly associated with breast cancer risk, but with the magnitude of the association slightly stronger for processed (risk increase per one SD increase in percent density (95 % CI): 1.55 (1.29, 1.85)) and “analogue-like” raw (1.52 (1.28, 1.80)) than for raw (1.35 (1.14, 1.60)) images. LIBRA percent density produced weaker associations with risk, albeit stronger for processed (1.32 (1.08, 1.61)) than raw images (1.17 (0.99, 1.37)). The percent density values yielded by the various density assessment/type of image combinations had similar ability to discriminate between patients and controls (area under the receiving operating curve values for percent density, age, BMI, parity and menopausal status combined ranged from 0.61 and 0.64).

**Conclusions:**

The findings showed that Cumulus can be used to measure density on all types of digital images. They also indicate that LIBRA may provide a valid fully automated alternative to the more labour-intensive Cumulus. However, the same digital image type and assessment method should be used when examining mammographic density across populations, or longitudinal changes in density within a single population.

**Electronic supplementary material:**

The online version of this article (doi:10.1186/s13058-016-0756-7) contains supplementary material, which is available to authorized users.

## Background

Mammographic density (MD) is one of the strongest risk factors for breast cancer [1, 2], and a major determinant of sensitivity to mammographic screening [3, 4]. The majority of research on MD has been conducted on digitised analogue films, with several approaches having been developed to measure MD on this type of image [5–13]. The semi-automated area-based method [6], as implemented by the Cumulus software, is currently regarded as the gold standard approach, as its measurements have been consistently associated with breast cancer risk [1].

Screen-film mammography is gradually being replaced by full-field digital mammography (FFDM). In contrast to the former, FFDM produces two types of image. Raw images (“for processing”) are initially captured but subsequently processed (“for presentation”) using manufacturer-specific algorithms (Additional file 1), which adjust the image contrast to improve diagnostic capability. The Cumulus software can be used on both raw and processed images, but as this algorithm was originally developed for digitised analogue images, special algorithms have been developed to convert raw digital images into “analogue-like” ones, i.e. into images that look like digitised analogue images [14]. However, the extent to which Cumulus density estimates vary depending on the type of digital image used is not clear. To our knowledge, only one small study (180 cases and 180 controls) has so far attempted to compare Cumulus MD estimates from digital raw images to those from processed images [15].

Cumulus is an observer-dependent and labour-intensive method, which precludes its routine use in clinical and screening settings. To overcome this, a novel and publicly available fully-automated area-based method - the Laboratory for Breast Radiodensity Assessment (LIBRA) - has been recently developed to quantify MD on both digital raw and processed images [16]. However, the extent to which LIBRA estimates from raw images are comparable to those from processed images is not known.

In this study, we compared the performance of Cumulus and LIBRA measurements from different types of digital images in a case-control study, by evaluating the extent to which they are associated with breast cancer risk factors, and by comparing their ability to predict breast cancer risk.

## Methods

### Study population

Details of the study population are described elsewhere [14]. Briefly, a case-control study was conducted between April 2010 and July 2012. Cases were women with newly diagnosed breast cancer seen in the Royal Marsden Hospital (RMS), London during the study period, who consented to take part in the study. Controls were recruited among cancer-free women who attended routine screening at the Central and East London Breast Screening Service (CELBSS) during the same period. CELBSS is part of the England and Wales Breast Screening Programme, a population-based programme which invites all women aged 47 (age 50 up to 2012) to 70 years (older women can self-refer) to attend mammographic screening once every 3 years. Women with a history of breast or ovarian cancer, or with breast implants, were excluded from the study. The study was approved by all relevant ethics committees (Research Ethics Committees from the Royal Marsden Hospital, the Barts and the London NHS Trust, and the London School of Hygiene and Tropical Medicine). Written informed consent was obtained from the participants.

### Data collection

Data on breast cancer risk factors were collected using a self-administered questionnaire at the time of screening for controls and after diagnostic confirmation for cases, and complemented with data from clinical records. Two-view (craniocaudal (CC) and mediolateral oblique (MLO)) FFDM was performed on each breast using Senographe DS units. Both raw and processed digital images were stored (Additional file 1: A and 1B, respectively), with the latter created using GE Healthcare algorithms.

Cumulus was originally developed for digitised analogue images. Thus, raw digital images were transformed so that they resembled as closely as possible that type of image. EasyScanConverter version 1.0 (Highnam Associated Limited) was used to convert the raw digital images into analogue-like images (Additional file 1: C) by simulating a film-like exposure (i.e. an automatic exposure control) and then mimicking a digitisation of that film using a high-end digitiser (i.e. Lumisys 85).

### Mammographic density measurements

MD readings were performed on anonymised images from the unaffected breast for cases and from a randomly selected breast for controls. As Cumulus readings are labour-intensive, only CC images were examined in the present study; this view was chosen over MLO because the latter is often affected by the superimposition of the pectoral muscle on the breast gland.

Cumulus (version 3) readings were performed on raw, processed and analogue-like CC images by a single reader (MCB) in batches of 250–300 images of the same type, with each batch comprising a similar proportion of cases and controls. To assess within-observer reliability each batch contained images of the same type from a 7 % random sample of all participants as duplicates. To compare between-observer reliability across different types of images, all (i.e. raw, processed and analogue-like) images from a random sample of 200 women were also read independently by another observer (IdSS). The two readers were kept blind to the characteristics of the women, including their case-control status. The fully automated LIBRA method (version 1.0.3, downloaded from https://www.cbica.upenn.edu/sbia/software/LIBRA/ on 20 December 2015) was also applied to both raw and processed images [16]. Both Cumulus and LIBRA are area-based methods and hence they provide areal estimates of breast size, absolute density and absolute non-density (all in cm^2^), and of percent density (PD).

### Statistical methods

The intra-class correlation coefficient (ICC) was used to examine within-observer and between-observer reliability in the Cumulus readings. Correlation between different approaches was assessed by estimating the Spearman correlation coefficient. Level of agreement between the measurements obtained by the different MD assessment method/image type combinations among controls was investigated by producing Bland-Altman plots. Quintile agreement was assessed as the proportion of women classified in the same quintile, or in the same ±1 adjacent quintile.

A square root transformation was used to normalise the area-based MD measurements. The association between each breast cancer risk factor and each density measure (i.e. PD, absolute density and non-density) was assessed among controls by linear regression models while adjusting for ethnicity (white versus other), age and body mass index (BMI) at mammography, ages at menarche and first birth, parity (nulliparous versus parous), menopausal status (premenopausal/perimenopausal versus postmenopausal), and ever-use of oral contraceptives and hormonal therapy (HT). Regression coefficients represent the change in each density measure (expressed in number of standard deviations (SD) on the transformed scale) associated with a unit change in the explanatory variable.

Logistic regression models were fitted to examine the association between density estimates and breast cancer risk, adjusting for age, BMI and the other variables mentioned previously. Density measurements were included in the models as continuous variables (in SD scores of the square root transformed values) or as quintiles or tertiles (defined among controls). To assess the robustness of the findings, sensitivity analyses were conducted by restricting the analysis to participants for whom density readings were available for all methods and to those aged <80 years. Multiple imputation methods were also used to impute values for women with missing confounder data. The ability of the various methods to discriminate between cases and controls was compared by estimating the area under the receiving operating curve (AUC). All analysis was performed in Stata 14.1.

## Results

**Fig. 1:**
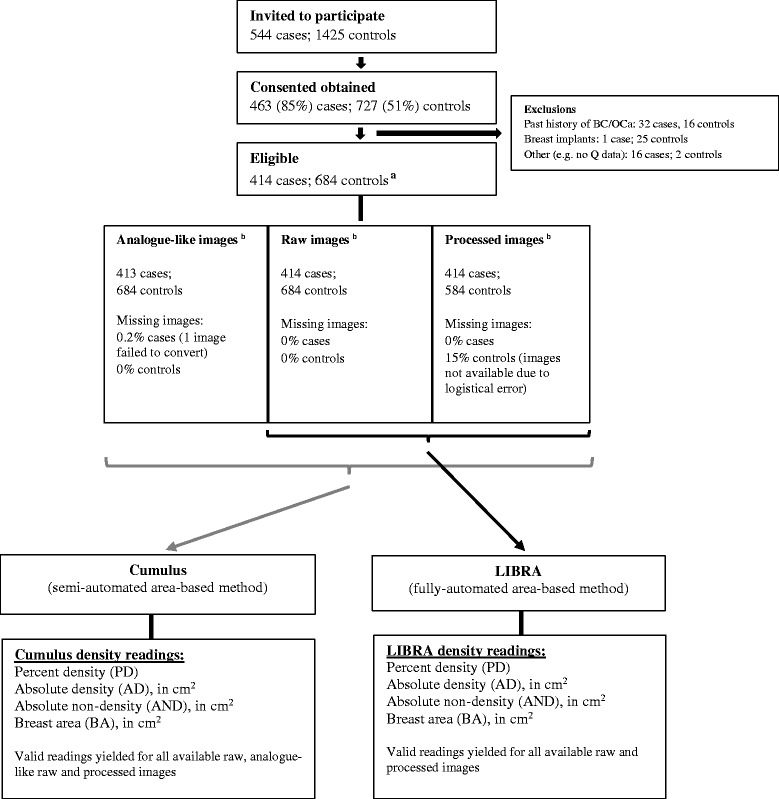
Flowchart detailing the recruitment and mammographic density assessment of the study participants. ^a^Only 684 controls were eligible for this study instead of the 685 included in the analysis reported by *Eng et al*., because no craniocaudal (CC) images were available for one control woman. ^b^CC image from the unaffected contralateral breast for cases and from a randomly selected breast for controls. *BC* breast cancer, *OCa* ovarian cancer, *Q* questionnaire

A total of 544 patients (cases) and 1425 controls were invited to participate. The response rate was 85 % for patients and 51 % for controls but only 414 patients and 684 controls were eligible (Fig. 1). Patients were older and more likely to be of white ethnicity than controls (Table 1). Raw images were available for all participants, analogue-like images were available for all participants except one patient (due to an image conversion error), and processed images were available for all patients but for only 85 % of the controls (n = 584) due to a logistical error (Fig. 1). Control women with missing and non-missing processed images did not differ in terms of their age (mean (SD): 59.5 (7.2) versus 59.4 (6.5) years, respectively), BMI (26.2 (5.8) versus 25.3 (4.8) kg/m^2^, respectively) or reproductive factors (e.g. percentage of parous women: 68.7 % versus 65.0 %, respectively). Both Cumulus and LIBRA produced readings for all available images (Fig. 1).

**Table 1:** Baseline characteristics of the participants, by case-control status

	Controls (*n* = 684)	Cases (*n* = 414)
Age, years, at mammography		
Mean (SD)	59.5 (6.6)	67.5 (12.7)
Missing, *n*	6	2
Ethnicity		
White, *n* (%)	519 (76.4)	370 (90.5)
Other, *n* (%)	160 (23.6)	39 (9.5)
Missing, *n*	5	5
Body mass index at mammography^a^, kg/m^2^		
Mean (SD)	26.1 (5.6)	26.4 (4.9)
Missing, *n*	29	46
Menopausal status at mammography^b^		
Premenopausal and perimenopausal, *n* (%)	91 (13.4)	55 (13.3)
Postmenopausal, *n* (%)	590 (86.6)	358 (86.7)
Missing, *n*	3	1
Ever use of oral contraceptives		
Yes, *n* (%)	208 (31.2)	143 (36.8)
No, *n* (%)	458 (68.8)	246 (63.2)
Missing, *n*	18	25
Ever use of hormonal therapy		
Yes, *n* (%)	197 (29.4)	175 (44.8)
No, *n* (%)	472 (70.6)	216 (55.2)
Missing, *n*	15	23
Parity		
Yes, *n* (%)	466 (69.0)	343 (84.0)
No, *n* (%)	209 (31.0)	65 (15.9)
Missing, *n*	9	6
Number of children^c^		
1 to 2, *n* (%)	300 (65.4)	218 (47.2)
3 to 4, *n* (%)	126 (27.5)	231 (50.0)
5+, *n* (%)	33 (7.2)	13 (2.8)
Missing, *n*	7	7

^a^Body mass index estimated form self-reported height and weight as weight/height^2^ (in kg/m^2^). ^b^Postmenopausal women defined as those who self-reported natural (i.e. cessation of menses for at least 12 months) or surgical menopause, were ≥55 years of age, or had ever used hormone therapy. Due to small numbers premenopausal (i.e. <55 years and still having regular periods) and perimenopausal (i.e. <55 years and having irregular periods) women were combined into a single category. ^c^Restricted to ever-parous women

### Comparisons between MD assessment method/type of image combinations

There was high within-observer reliability in the Cumulus readings for all three types of images (e.g. the ICC for Cumulus PD was 0.97, 0.91 and 0.90 for raw, processed and analogue-like images, respectively). Inter-observer reliability was also high among the random sample of 200 women whose images were independently read by a second observer (the ICC for Cumulus PD was 0.89, 0.90 and 0.83 for raw, processed and analogue-like images, respectively).

**Fig. 2:**
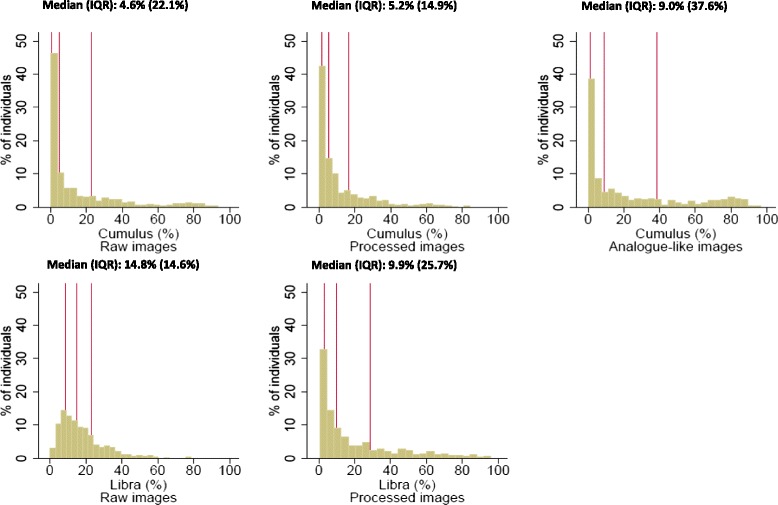
Distribution of percent density (*PD*) values yielded by Cumulus and Laboratory for Breast Radiodensity Assessment (*Libra*) on different types of digital images in control women

The PD measurements yielded by Cumulus on the three types of images were highly correlated (Spearman rank correlation coefficient (*r*) ≥0.80, *P* < 0.0001 for all; Table 2). The distributions for Cumulus PD and for absolute density, absolute non-density and breast size are shown in Fig. 2 and Additional files 2, 3, and 4). The PD distributions were all right-skewed (Fig. 2), with a high proportion of women having no measurable or very low PD (approximately 50 % of women had PD <10), but with the PD distribution for processed images being much narrower (interquartile range (IQR) 14.8 %) than those for raw (IQR 22.0 %) or analogue-like images (IQR 38.0 %) (Fig. 2). However, the Bland-Altman plots (Additional file 5) showed no systematic differences (after applying a square root transformation to normalise the distributions) between the Cumulus PD values produced by the three types of images, as the mean of their differences was close to zero, and no evidence of a trend in the mean difference with increasing average PD; there was, however, high variability in the measurements yielded by the three types of images, particularly so for those close to the mean of the average PD values. In all, between 52 % and 62 % of women were assigned to the same PD quintile, and >92 % assigned to the same PD quintile ± 1 by Cumulus on the three types of images (Additional file 6).

**Table 2:** Spearman’s rank correlation coefficients between the percent density estimates yielded by the various density assessment method/image type combinations in control women

Method	Image type	Area-based methods				
		Cumulus			LIBRA	
		Raw	Processed	Analogue-like	Raw	Processed
Cumulus	Raw	-	0.85* (*n* = 584)	0.81* (*n* = 684)	0.65* (*n* = 684)	0.75* (*n* = 584)
	Processed	-	-	0.80* (*n* = 584)	0.66* (*n* = 584)	0.74* (*n* = 584)
	Analogue-like	-	-		0.59* (*n* = 684)	0.71* (*n* = 584)
LIBRA	Raw	-	-			0.71* (*n* = 584)
	Processed	-	-		-	-

*P < 0.0001; *n* number of control women on which the analysis was based. *LIBRA* Laboratory for Breast Radiodensity Assessment

LIBRA PD measurements on raw and processed images among control women were strongly correlated (*r* = 0.71, *P* < 0.0001) (Table 2), despite the distributions having a different shape. The distribution of LIBRA PD values from raw images was narrower than that from processed images (IQR 14.6 % and 25.7 %, respectively; Fig. 2), with the latter yielding higher proportions of women with very low PD values and of women with PD > 40 %. Nevertheless, the Bland-Altman plot showed no systematic differences in the square root transformed LIBRA PD values from the two types of images (Additional file 7). In all, 47 % of women were assigned to the same quintile, and 64 % to the same quintile ±1 of LIBRA PD by both raw and digital images (Additional file 6).

Cumulus and LIBRA PD measurements taken on the same type of image were moderately to strongly correlated (r = 0.65 and *r* = 0.74 for raw and processed images, respectively; *P* < 0.0001 for both; Table 2). In particular, LIBRA readings on raw images tended to overestimate PD at the lower end, but to underestimate PD at the upper end of the PD distribution relative to Cumulus on the same type of image (Fig. 2). However, Bland-Altman plots showed no systematic differences in square root transformed Cumulus and LIBRA PD values from the same type of image (Additional file 7). In all, 45–47 % of women were assigned to the same quintile and 81–87 % to the same ±1 quintile by LIBRA and Cumulus PD estimates on the same type of image (Additional file 6). Interestingly, however, the level of agreement between LIBRA PD measurements in raw and LIBRA PD in processed images was not high (only 47 % and 64 % of women were assigned, respectively, to the same quintile or the same ±1 quintile by LIBRA raw and processed readings).

### Association with breast cancer risk factors among controls

**Fig. 3:**
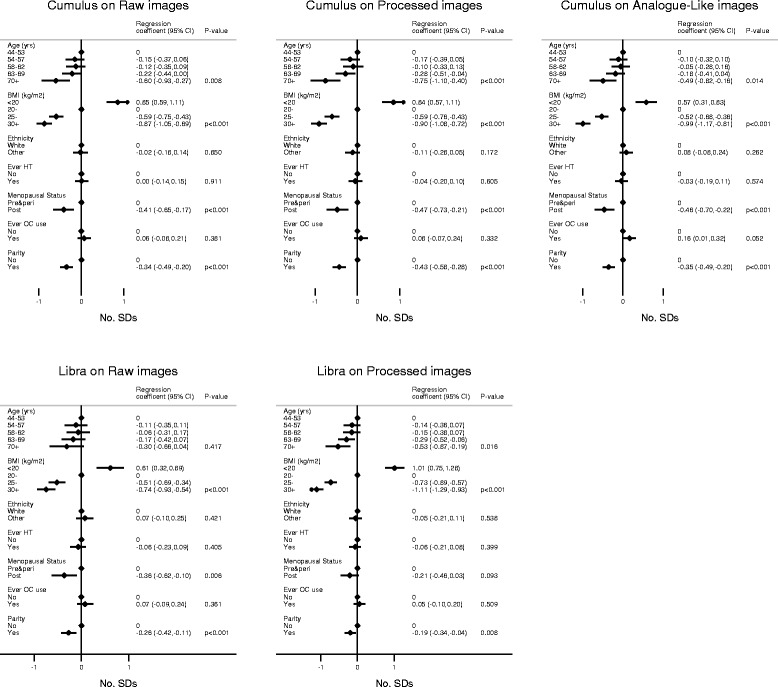
Mutually adjusted associations between known determinants of mammographic density and percent density (*PD*) readings in control women. *P value* is *P* for linear trend. *No. SDs* is the number of standard deviations (on the square root transformed scale) *BMI* body mass index, *HT* hormonal therapy, *OC* oral contraceptives, *Libra* Laboratory for Breast Radiodensity Assessment

Age was inversely associated with Cumulus PD, albeit more markedly for processed images (Fig. 3), reflecting negative associations with absolute density and positive associations with absolute non-density (Additional files 8 and 9). A similar inverse association with age was observed for LIBRA PD, driven mainly by a positive association between age and absolute non-density and, for processed images only, also by an inverse association with absolute density, as LIBRA absolute density on raw images was not associated with age (Additional files 8 and 9).

Cumulus PD was strongly inversely associated with BMI regardless of the type of image used (Fig. 3), driven by both a negative association of this variable with absolute density and a positive association with absolute non-density (Additional files 8 and 9). A similar negative association was observed between BMI and LIBRA PD on raw and processed images, driven mainly by strong positive associations with absolute non-density and, for processed images only, also by a negative association with absolute density, as LIBRA absolute density on raw images was not associated with BMI (Additional files 8 and 9).

Regardless of type of image used, both Cumulus and LIBRA PD were lower in postmenopausal women, reflecting mainly decreases in absolute density, and in parous women, driven by lower absolute density and higher absolute non-density among these women relative to their nulliparous counterparts. There were no clear associations between Cumulus or LIBRA PD and ethnicity, ever-use of oral contraceptives (except for a positive association with Cumulus PD on analogue-like images) or hormonal therapy (Fig. 3), or ages at menarche or first birth (data not shown).

### Breast cancer risk

**Fig. 4:**
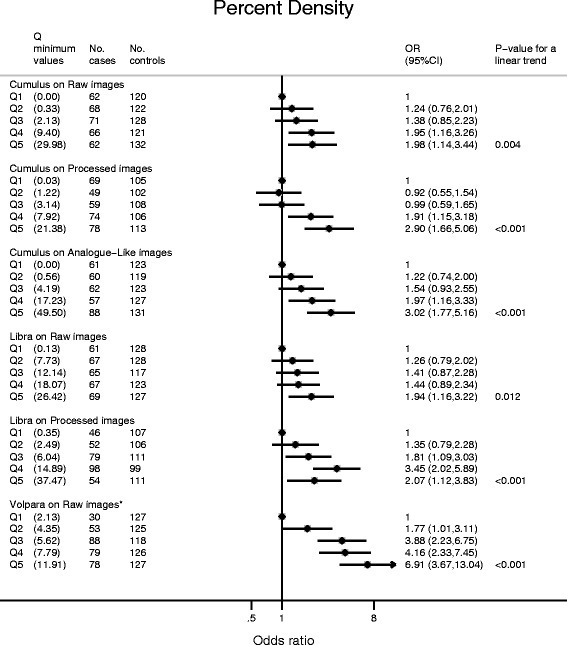
Breast cancer risk by quintiles of percent density (PD) for each density assessment method/type of image combination. Quintiles (*Q*) of the PD distribution among controls. Odds ratios (*OR*) and 95 % CI as estimated by logistic regression models adjusted for age, body mass index (BMI), menopausal status, parity, age at menarche, ever-use of oral contraceptives and hormonal therapy. *Libra* Laboratory for Breast Radiodensity Assessment

Cumulus PD was positively associated with breast cancer risk for all types of images (Fig. 4). Women in the top quintile had 3.02 (95 % CI 1.77, 5.16), 2.90 (1.66, 5.06) and 1.98 (1.14, 3.44) times the risk of those in the bottom one for analogue-like, processed and raw images, respectively (*P* for linear trend ≤0.004 for all). The positive associations between Cumulus PD and risk reflected positive associations between absolute density and risk, and negative associations between absolute non-density and risk (Additional file 10). Similar positive associations were observed between LIBRA PD and breast cancer risk for raw and processed images (odds ratio (OR) (95 % CI) for the top PD quintile versus the lowest PD quintile: 1.94 (1.16, 3.22) and 2.07 (1.12, 3.83), respectively) (Fig. 4) which, for processed images only, was mainly driven by a positive association between absolute density and risk (Additional file 10). PD values in the two bottom quintiles were low (Fig. 4), but analysis by tertiles revealed similar patterns (e.g. OR (95 % CI) for top third versus bottom third: 1.86 (1.22, 2.82) for Cumulus raw, 2.58 (1.67, 3.99) for Cumulus processed, 2.16 (1.42, 3.27) for Cumulus analogue-like, 1.86 (1.22, 2.82) for LIBRA raw, and 2.23 (1.40, 3.55) for LIBRA processed images.

Risk increases per one SD increase in Cumulus PD were slightly higher for processed (OR 1.55; 95 % CI 1.29, 1.85) and analogue-like images (1.52; 1.28, 1.80) than for raw images (1.35; 1.14, 1.60) (Table 3). Weaker associations were observed for LIBRA PD on processed (1.32; 1.08, 1.61) and raw images (1.17; 0.99, 1.37) (Table 3). The risk increases associated with one SD increase in absolute density were similar to those associated with an equivalent increase in PD for both Cumulus and LIBRA regardless of type of images, except for LIBRA on raw images, for which the association with absolute density was weaker and no longer statistically significant (Table 3). The results of sensitivity analyses were similar (Table 3).

**Table 3:** Cumulus and LIBRA mammographic density measurements and breast cancer risk, by type of digital image

	Cumulus						LIBRA			
	Raw images		Processed images		Analogue-like images		Raw images		Processed images	
	*N*	OR^a^ (95 % CI)	*N*	OR^a^ (95 % CI)	*N*	OR^a^ (95 % CI)	*N*	OR^a^ (95 % CI)	*N*	OR^a^ (95 % CI)
Percent density (%)										
All readings^b^	952	1.35 (1.14,1.60)	863	1.55 (1.29,1.85)	951	1.52 (1.28,1.80)	952	1.17 (0.99,1.37)	863	1.32 (1.08,1.61)
Multiple imputation^b,c^	1098	1.58 (1.46,1.70)	998	1.91 (1.76,2.08)	1097	1.93 (1.78,2.09)	1098	1.21 (1.12,1.32)	998	1.65 (1.49,1.82)
Restricted to women aged <80 years^b^	899	1.35 (1.13,1.60)	811	1.54 (1.28,1.85)	898	1.54 (1.29,1.83)	899	1.18 (1.01,1.39)	811	1.29 (1.06,1.58)
Restricted to women with data for all MD assessment/type of image approaches^b^	814	1.30 (1.07,1.58)	814	1.47 (1.21,1.78)	814	1.47 (1.22,1.78)	862	1.18 (1.00,1.39)	862	1.32 (1.08,1.61)
Dense area (in cm^2^)										
All readings^b^	952	1.34 (1.15,1.56)	863	1.53 (1.30,1.79)	951	1.45 (1.24,1.68)	952	1.10 (0.96,1.27)	863	1.39 (1.17,1.64)
Multiple imputation^b,c^	1098	1.47 (1.38,1.58)	998	1.73 (1.61,1.87)	1097	1.65 (1.54,1.77)	1098	0.99 (0.92,1.06)	998	1.56 (1.42,1.71)
Restricted to women aged <80 years^b^	899	1.33 (1.14,1.55)	811	1.52 (1.30,1.78)	898	1.46 (1.26,1.70)	899	1.11 (0.97,1.28)	811	1.36 (1.14,1.61)
Restricted to women with MD data for all MD assessment/type of image approaches^b^	814	1.30 (1.10,1.54)	814	1.46 (1.24,1.73)	814	1.43 (1.21,1.68)	862	1.11 (0.96,1.28)	862	1.38 (1.17,1.64)
Non-dense area (in cm^2^)										
All readings^b^	952	0.75 (0.61,0.91)	863	0.75 (0.61,0.93)	951	0.65 (0.53,0.79)	952	0.83 (0.67,1.02)	863	0.84 (0.67,1.05)
Multiple imputation^b,c^	1098	0.57 (0.52,0.61)	998	0.49 (0.45,0.54)	1097	0.45 (0.41,0.49)	1098	0.61 (0.56,0.68)	998	0.54 (0.49,0.60)
Restricted to women aged <80 years^b^	899	0.75 (0.61,0.91)	811	0.74 (0.60,0.92)	898	0.64 (0.52,0.78)	899	0.82 (0.66,1.01)	811	0.84 (0.67,1.06)
Restricted to women with MD data for all MD assessment/type of image approaches^b^	814	0.78 (0.63,0.98)	814	0.78 (0.62,0.98)	814	0.70 (0.56,0.88)	862	0.82 (0.66,1.01)	862	0.84 (0.67,1.05)

^a^Odds ratios (OR) represent the change in breast cancer risk associated with one standard deviation (SD) increase in percent density, absolute density and absolute non-density associated with each one of the mammographic density (MD) assessment method/type of image combination. OR and 95 % CI estimated by logistic regression models based on standardised values of square root transformed density measurements (adjusted for age, body mass index, menopausal status, parity, age at menarche, ever-use of oral contraceptive and hormonal therapy). ^b^Based on density measurements taken from the unaffected breast in patients (cases) and a randomly selected breast (left or right) in controls. ^c^Multiple imputation methods used to impute values for women with missing information on age, body mass index, menopausal status and/or parity

The PD values yielded by the various mammographic density (MD) assessment method/type of image combinations had a similar ability to discriminate between cases and controls of screening age, i.e. aged 50–69 years. AUC values for PD, age, BMI, parity and menopausal status ranged from 0.61 and 0.64; similar findings were observed for absolute density (Additional file 11).

## Discussion

This study aimed to compare the performance of two area-based methods - Cumulus and LIBRA - on different types of FFDM images. The findings showed that Cumulus density measurements had high within-observer and between-observer reliability regardless of the type of digital image used. Processed images are, by definition, processed to enhance the visibility of breast features, but we found little difference on average between the Cumulus PD estimates yielded from raw and processed images, which is consistent with a previous study [15]. All three types of images produced positive associations between PD and breast cancer risk; these associations were, however, stronger for Cumulus on analogue-like and processed images than for Cumulus on raw images.

One previous study [15], based on a smaller numbers of cases (i.e. 180 only), has also shown that area-based density estimates from processed digital images are equally good predictors of breast cancer risk as those from raw images. However, our findings indicate that the distributions of the Cumulus readings yielded by the three types of images are not equivalent. Thus, the same type of image should be used when comparing Cumulus MD across different populations, or when assessing longitudinal changes in Cumulus MD within a single population. If this is not possible, Cumulus measurements on different types of images should be obtained for a representative subset of participants, to allow calibration of all Cumulus measurements to a single type of image.

We also examined LIBRA, a fully-automated area-based method, which was specifically developed for use on both raw and processed digital images. Associations of known breast cancer risk factors with LIBRA PD were slightly weaker than those with Cumulus PD, and so was the association between LIBRA PD measurements and breast cancer risk. A previous case-control study in the USA, based on a smaller sample size (106 cases and 318 controls), reported stronger association between LIBRA PD on raw images and risk (OR per one SD increase in PD (on a logarithmic scale) adjusted for the Gail risk factors: 1.64; 95 % CI 1.25, 2.14) [17] than the one observed in this study (1.32; 1.08, 161; Table 3). The present study comprised a higher proportion of women with low PD (50 % of control women had Cumulus PD <5 % as assessed on raw and processed images; median LIBRA PD of 14.8 % (IQR 14.6 %) versus a mean of 27 % (SD = 14.7 %) in the USA study [17]), in line with their relatively old age and postmenopausal status at mammography, and LIBRA may perform less well in low-density images. Nevertheless, our findings are consistent with LIBRA being a valid fully-automated alternative to Cumulus, which could be used in both processed and raw images. Further assessments of LIBRA are required, preferably in study populations with a larger number of cases and access to images taken several years prior to breast cancer diagnosis.

It is noteworthy that the area-based Cumulus and LIBRA approaches examined here produced weaker associations between PD and breast cancer risk than the fully automated volumetric VOLPARA algorithm. The latter was examined in a previous analysis of data from this case-control study [14] (OR per one SD increase in VOLPARA PD is equal to 1.75 (95 % CI 1.45, 2.10) for the subset of images used in the present study). However, VOLPARA can only be used on raw images; this is a major limitation as most clinical and screening settings only save processed images due to picture archive and communication system (PACS) space constraints. The development of MD assessment methods for use on processed images has been hampered by the fact that each manufacturer develops its own algorithm for converting raw images into processed images - often kept undisclosed - and this may compromise the comparability of MD measurements made on processed images from different manufacturers. We were not able to compare density measurements across different manufacturers as all images included in this study were processed according to GE Healthcare algorithms.

Consistently with other studies [12, 18], the AUC estimates for the density measurements yielded by the various method/type of image combinations (jointly with age, BMI and reproductive-related factors) were low (between 61 % and 65 %) - albeit similar to those yielded by current prediction models such as the Gail model - and therefore, of little value for individual risk prediction.

Strengths of this study include the availability of three different types of digital images taken from the same women at the same point in time, and the collection of risk factor data close to the time of mammography. All Cumulus measurements were made by a single observer, blinded to the characteristics of the participants including their case-control status; in addition, we were able to compare within-observer and between-observer reliability of the Cumulus MD measurements across the three types of images.

Limitations include the fact that the majority of participants had low PD, consistent with their age and postmenopausal status; hence, the extent to which the findings can be generalised to younger women is not known. The response rate was lower for healthy controls compared to cases (51 % versus 85 %) but any potential bias is likely to have affected all MD assessment method/type of image combinations examined similarly. Data on breast cancer risk factors were self-reported but as density is not routinely assessed in the UK, and hence women are not informed about it, any misclassification is likely to have been non-differential. Processed images were missing for 15 % of the control participants due to a logistical error; however, no differences were observed between women with missing images and those for whom processed images were available, in terms of the distribution of breast cancer risk factors.

## Conclusions

This study demonstrated that Cumulus can be used to produce valid MD estimates from raw, analogue-like and processed digital images. Although the Cumulus measurements yielded by the three types of images were strongly correlated, their distributions were not equivalent. Nevertheless, Cumulus PD estimates were all strongly associated with breast cancer risk regardless of the type of image used, but with the magnitude of this association being slightly stronger for analogue-like and processed images than for raw images. The findings also provide further evidence that LIBRA on processed images may be a valid fully automated alternative to the more labour-intensive Cumulus approach. The associations with breast cancer risk for LIBRA measurements on raw images were weaker than those produced by Cumulus. Nevertheless, LIBRA may be the only feasible option in large-scale studies based on raw images for which it will be prohibitively expensive and time-consuming to use Cumulus, with the weaker association between PD and breast cancer risk being offset by a much larger sample size. The findings also highlighted the need to use the same type of image when comparing MD measurements across populations, or when evaluating longitudinal changes in MD within a single population.

## Additional files

Additional file 1: Different types of digital images: raw (A), processed (B) and “analogue-like” raw (C). (PDF 46 kb) Additional file 2: Distribution of absolute density (dense area) values yielded by Cumulus and LIBRA on different types of digital images in control women. (PDF 114 kb) Additional file 3: Distribution of absolute non-density (non-dense area) values yielded by Cumulus and LIBRA on different types of digital images in control women. (PDF 114 kb) Additional file 4: Distribution of the control participants by breast size (area) values yielded by Cumulus and LIBRA on different types of digital images. (PDF 123 kb) Additional file 5: Bland-Altman plots assessing agreement between Cumulus percent density measurements (in SD scores of the square root transformed values) yielded by different types of images: 95 % limits of agreement = mean difference ± 1.96 SD. The *grey regression line* represents the proportion change in difference in percent density estimates for a unit increase in average percent density (and the *grey area* around it the 95 % confidence intervals of the regression coefficient). (PDF 246 kb) Additional file 6: Quintile agreement in the percent density estimates yielded by the various density assessment method/image type combinations in control women. (DOCX 16 kb) Additional file 7: Bland-Altman plots assessing agreement between Cumulus and LIBRA percent density measurements on raw and processed images, and between LIBRA percent density measurements on raw and processed images (in SD scores of the square root transformed values): 95 % limits of agreement = mean difference ± 1.96 SD. The *grey regression line* represents the proportion change in difference in percent density estimates for a unit increase in average percent density (and the *grey area* around it the 95 % confidence intervals of the regression coefficient). (PDF 248 kb) Additional file 8: Mutually-adjusted associations of known determinants of mammographic density with absolute density readings in control women. *BMI* body mass index, *HT* hormonal therapy, *OC* oral contraceptives, *P-value P* for linear trend, *No. SDs* number of standard deviations (on the transformed scale). (PDF 63 kb) Additional file 9: Mutually-adjusted associations of known determinants of mammographic density with absolute non-density readings in control women. *BMI* body mass index, *HT* hormonal therapy, *OC* oral contraceptives, *P-value P* for linear trend, *No. SDs* number of standard deviations (on the square root transformed scale). (PDF 63 kb) Additional file 10: Breast cancer risk by quintiles of absolute density and absolute non-density for each density assessment method/type of image combination. Quintiles of the absolute density and absolute non-density distributions among controls. Odds ratios (*OR*) and 95 % CI as estimated by logistic regression models adjusted for age, body mass index (BMI), menopausal status, parity, age at menarche, ever-use of oral contraceptives and hormonal therapy. (PDF 116 kb) Additional file 11: Area under the receiving operating curve (*AUC*) for percent and absolute density for each density assessment method/type of image combination. (DOCX 14 kb)

## Abbreviations

AUC: area under the curve
BMI: body mass index
CC: craniocaudal
CI: confidence interval
FFDM: full-field digital mammography
HT: hormonal therapy
ICC: intra-class correlation coefficient
IQR: inter-quartile range
LIBRA: Laboratory for Breast Radiodensity Assessment
MD: mammographic density
MLO: mediolateral
OC: oral contraceptives
OR: odds ratio
PD: percent density
*r*: Spearman rank correlation coefficient
SD: standard deviation
