# Supplementary figures and images for: Impact of type of full-field digital image on mammographic density assessment and breast cancer risk estimation: a case-control study
Source: Breast Cancer Res. 2016 Sep 26;18:96. doi: 10.1186/s13058-016-0756-7 (PMC5037867; doi:10.1186/s13058-016-0756-7)

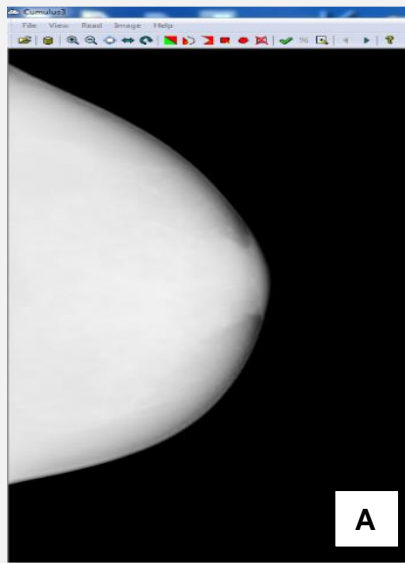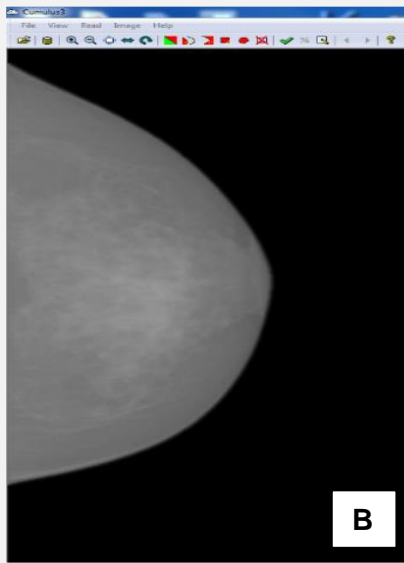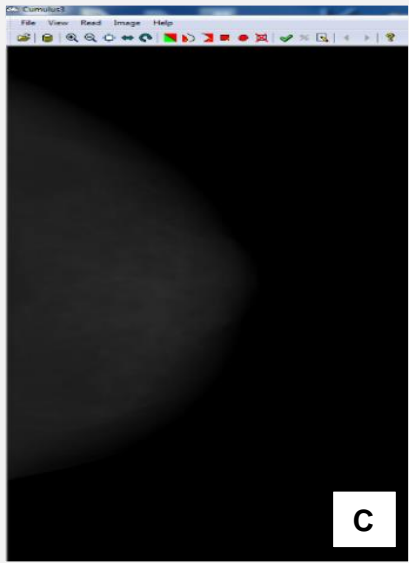

Supplement: Additional file 1: — Different types of digital images: raw (A), processed (B) and “analogue-like” raw (C). (PDF 46 kb) [file 13058_2016_756_MOESM1_ESM.pdf]

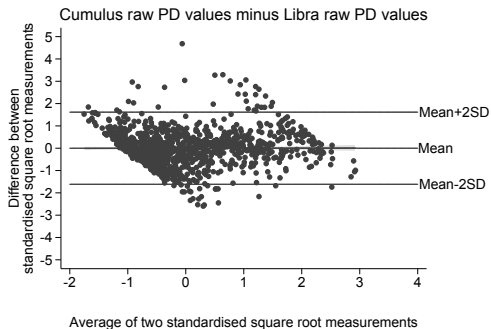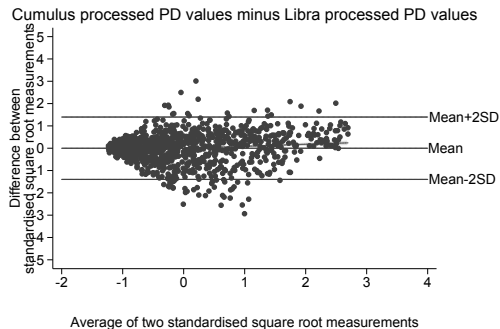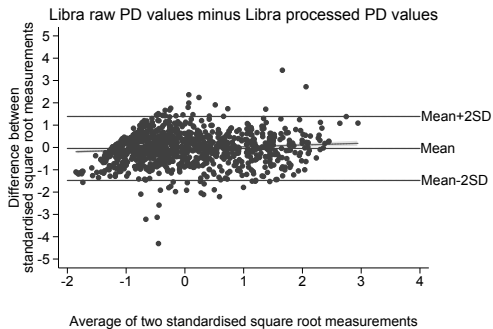

Supplement: Additional file 7: — Bland-Altman plots assessing agreement between Cumulus and LIBRA percent density measurements on raw and processed images, and between LIBRA percent density measurements on raw and processed images (in SD scores of the square root transformed values): 95 % limits of agreement = mean difference ± 1.96 SD. The grey regression line represents the proportion change in difference in percent density estimates for a unit increase in average percent density (and the grey area around it the 95 % confidence intervals of the regression coefficient). (PDF 248 kb) [file 13058_2016_756_MOESM7_ESM.pdf]

## Dense area

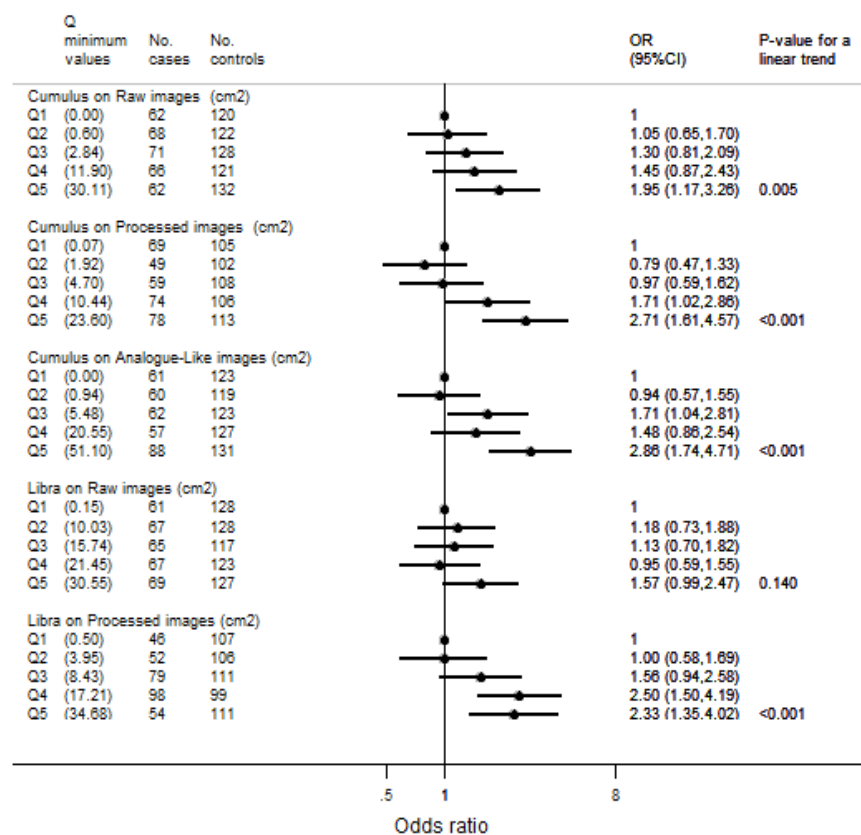

## Non-dense area

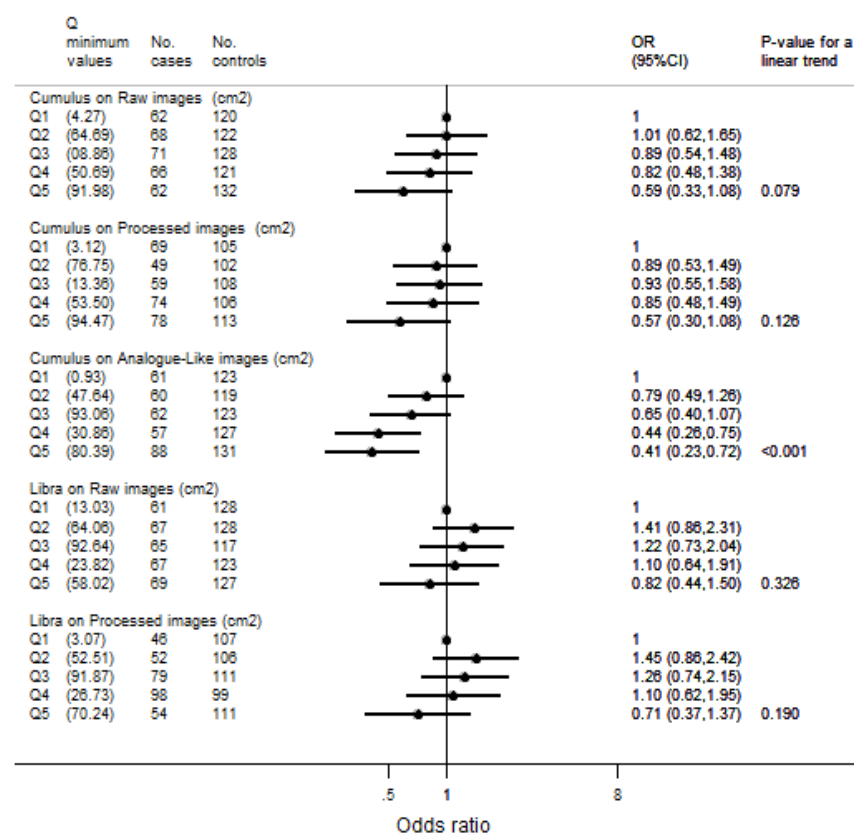

Supplement: Additional file 10: — Breast cancer risk by quintiles of absolute density and absolute non-density for each density assessment method/type of image combination. Quintiles of the absolute density and absolute non-density distributions among controls. Odds ratios (OR) and 95 % CI as estimated by logistic regression models adjusted for age, body mass index (BMI), menopausal status, parity, age at menarche, ever-use of oral contraceptives and hormonal therapy. (PDF 116 kb) [file 13058_2016_756_MOESM10_ESM.pdf]
