# Supplementary material for: Impact of type of full-field digital image on mammographic density assessment and breast cancer risk estimation: a case-control study
Source: Breast Cancer Res. 2016 Sep 26;18:96. doi: 10.1186/s13058-016-0756-7 (PMC5037867; doi:10.1186/s13058-016-0756-7)
Supplement: Additional file 2: — Distribution of absolute density (dense area) values yielded by Cumulus and LIBRA on different types of digital images in control women. (PDF 114 kb) [file 13058_2016_756_MOESM2_ESM.pdf]

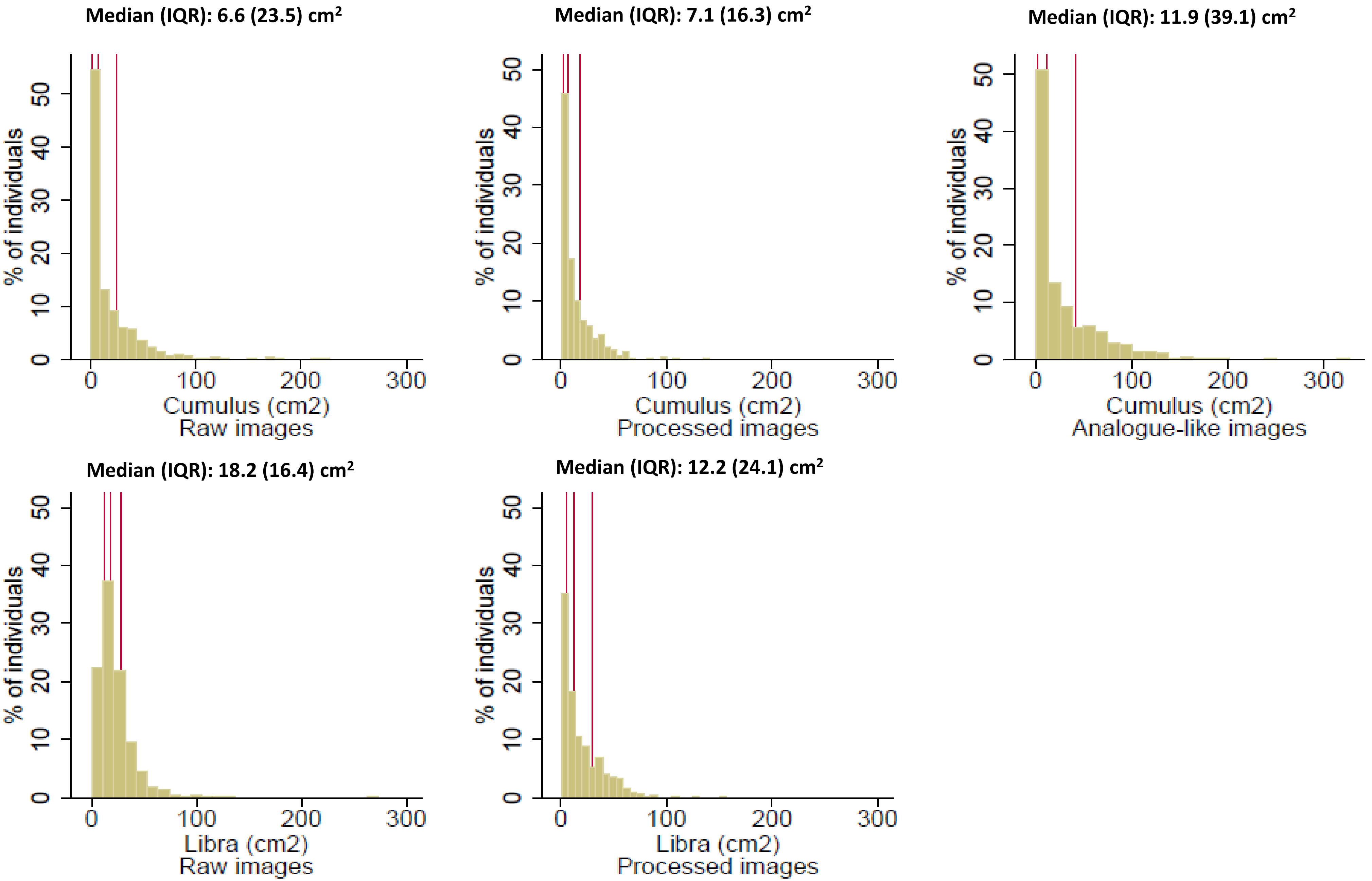

Median (IQR): 6.6 (23.5) cm^2
% of individuals
Cumulus (cm2)
Raw images
Median (IQR): 7.1 (16.3) cm^2
% of individuals
Cumulus (cm2)
Processed images
Median (IQR): 11.9 (39.1) cm^2
% of individuals
Cumulus (cm2)
Analogue-like images
Median (IQR): 18.2 (16.4) cm^2
% of individuals
Libra (cm2)
Raw images
Median (IQR): 12.2 (24.1) cm^2
% of individuals
Libra (cm2)
Processed images
