# Supplementary material for: Impact of type of full-field digital image on mammographic density assessment and breast cancer risk estimation: a case-control study
Source: Breast Cancer Res. 2016 Sep 26;18:96. doi: 10.1186/s13058-016-0756-7 (PMC5037867; doi:10.1186/s13058-016-0756-7)
Supplement: Additional file 3: — Distribution of absolute non-density (non-dense area) values yielded by Cumulus and LIBRA on different types of digital images in control women. (PDF 114 kb) [file 13058_2016_756_MOESM3_ESM.pdf]

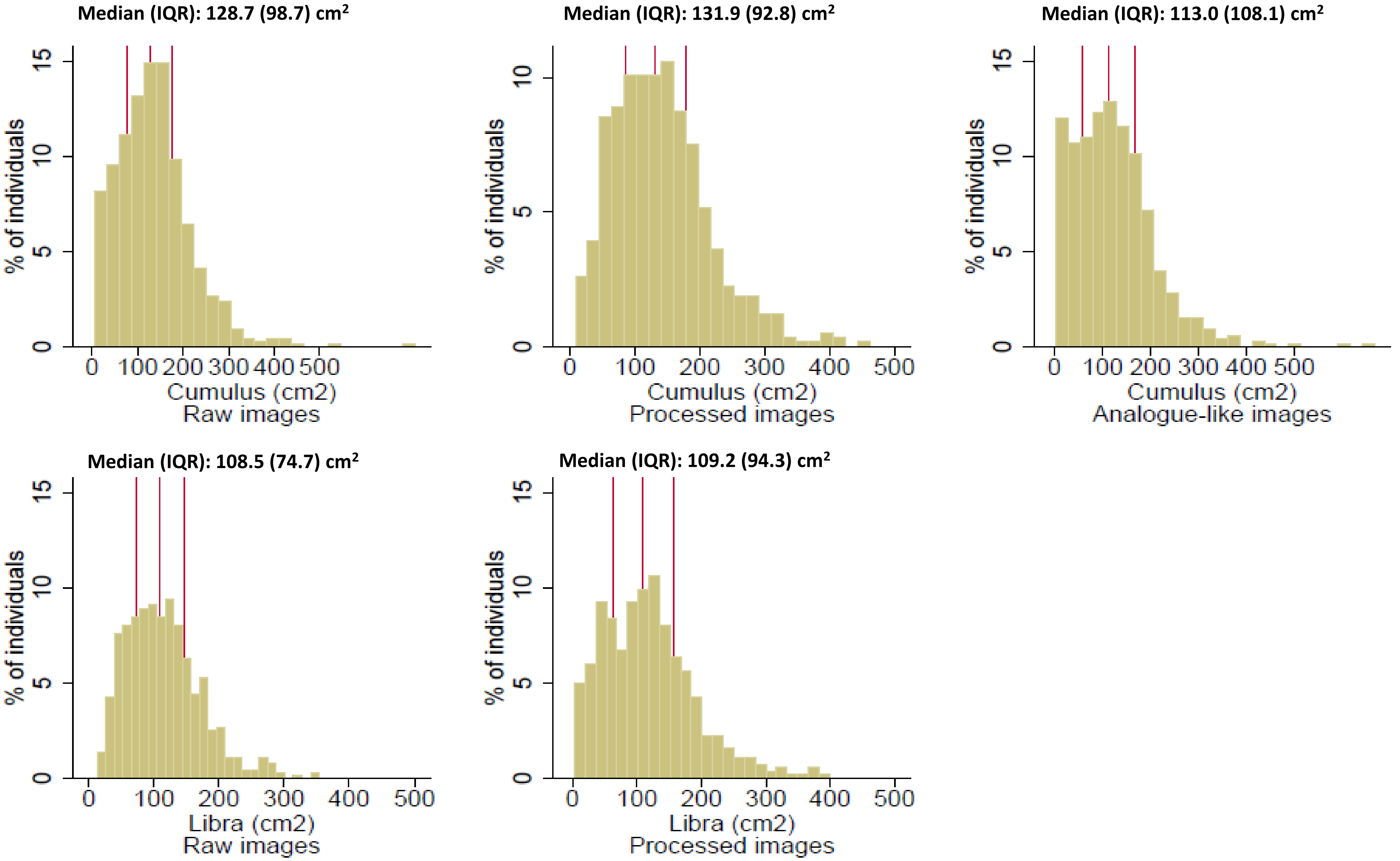
Median (IQR): 128.7 (98.7) cm²
% of individuals
Cumulus (cm2)
Raw images
Median (IQR): 131.9 (92.8) cm²
% of individuals
Cumulus (cm2)
Processed images
Median (IQR): 113.0 (108.1) cm²
% of individuals
Cumulus (cm2)
Analogue-like images
Median (IQR): 108.5 (74.7) cm²
% of individuals
Libra (cm2)
Raw images
Median (IQR): 109.2 (94.3) cm²
% of individuals
Libra (cm2)
Processed images
