# Supplementary material for: Impact of type of full-field digital image on mammographic density assessment and breast cancer risk estimation: a case-control study
Source: Breast Cancer Res. 2016 Sep 26;18:96. doi: 10.1186/s13058-016-0756-7 (PMC5037867; doi:10.1186/s13058-016-0756-7)
Supplement: Additional file 4: — Distribution of the control participants by breast size (area) values yielded by Cumulus and LIBRA on different types of digital images. (PDF 123 kb) [file 13058_2016_756_MOESM4_ESM.pdf]

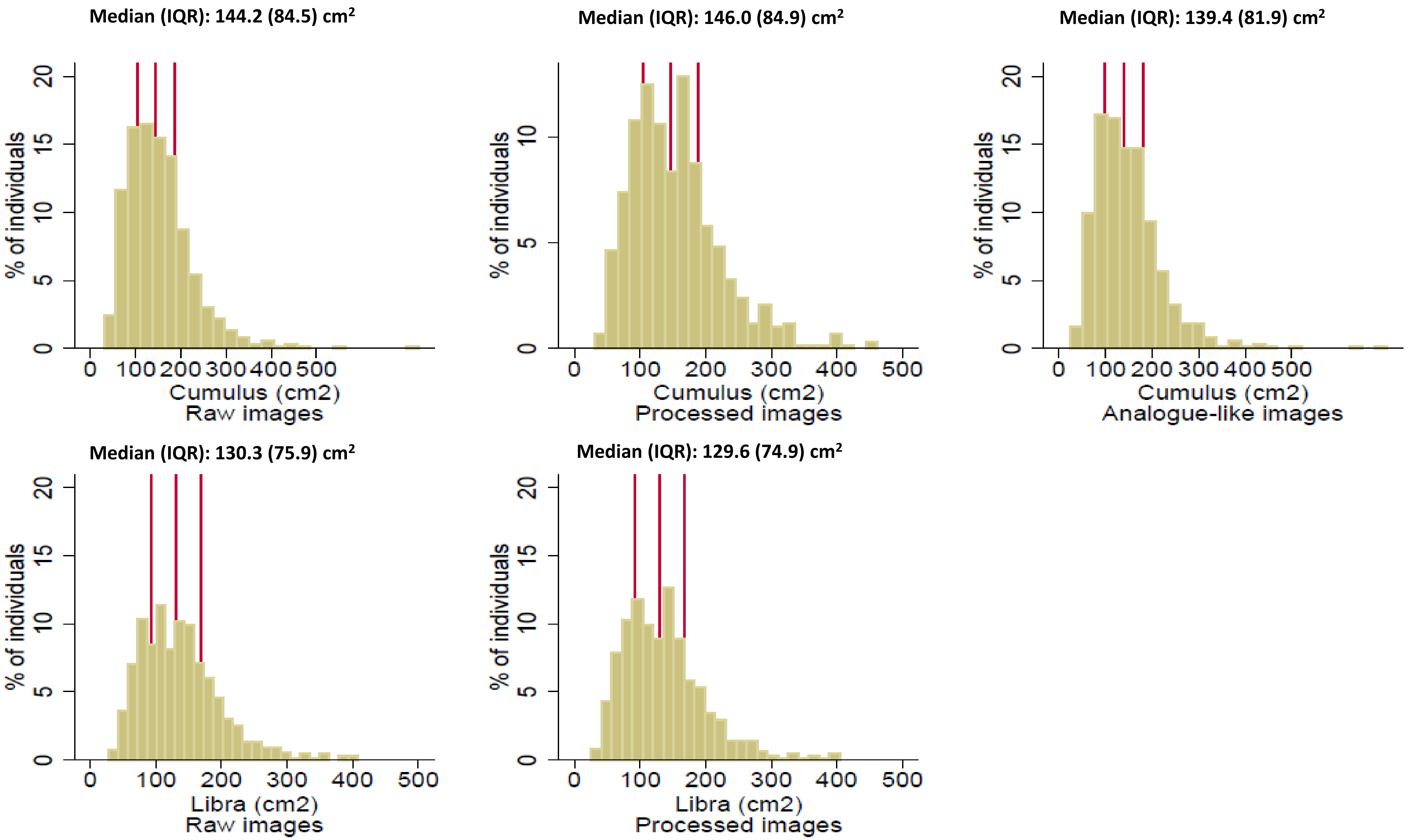
Median (IQR): 144.2 (84.5) cm²
% of individuals
0 5 10 15 20
0 100 200 300 400 500
Cumulus (cm2)
Raw images
Median (IQR): 146.0 (84.9) cm²
% of individuals
0 5 10
0 100 200 300 400 500
Cumulus (cm2)
Processed images
Median (IQR): 139.4 (81.9) cm²
% of individuals
0 5 10 15 20
0 100 200 300 400 500
Cumulus (cm2)
Analogue-like images
Median (IQR): 130.3 (75.9) cm²
% of individuals
0 5 10 15 20
0 100 200 300 400 500
Libra (cm2)
Raw images
Median (IQR): 129.6 (74.9) cm²
% of individuals
0 5 10 15 20
0 100 200 300 400 500
Libra (cm2)
Processed images
