# Supplementary material for: Impact of type of full-field digital image on mammographic density assessment and breast cancer risk estimation: a case-control study
Source: Breast Cancer Res. 2016 Sep 26;18:96. doi: 10.1186/s13058-016-0756-7 (PMC5037867; doi:10.1186/s13058-016-0756-7)
Supplement: Additional file 5: — Bland-Altman plots assessing agreement between Cumulus percent density measurements (in SD scores of the square root transformed values) yielded by different types of images: 95 % limits of agreement = mean difference ± 1.96 SD. The grey regression line represents the proportion change in difference in percent density estimates for a unit increase in average percent density (and the grey area around it the 95 % confidence intervals of the regression coefficient). (PDF 246 kb) [file 13058_2016_756_MOESM5_ESM.pdf]

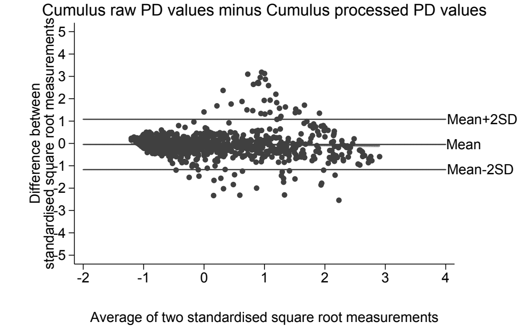
Cumulus raw PD values minus Cumulus processed PD values
Difference between standardised square root measurements
Mean+2SD
Mean
Mean-2SD
Average of two standardised square root measurements

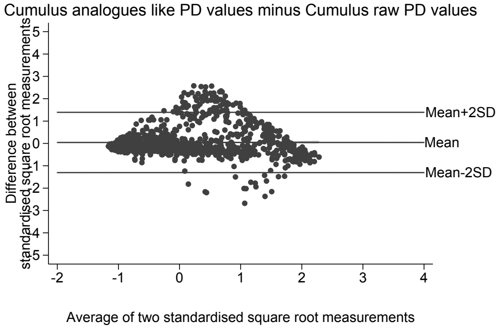
Cumulus analogues like PD values minus Cumulus raw PD values
Difference between standardised square root measurements
Mean+2SD
Mean
Mean-2SD
Average of two standardised square root measurements

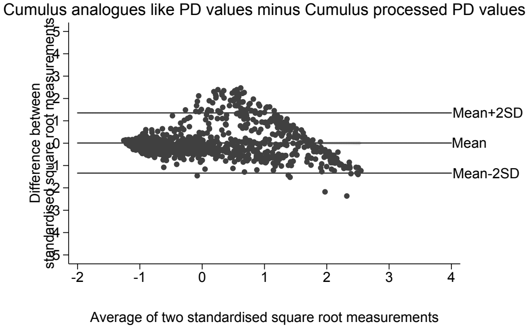
Cumulus analogues like PD values minus Cumulus processed PD values
Difference between standardised square root measurements
Mean+2SD
Mean
Mean-2SD
Average of two standardised square root measurements
