# Supplementary material for: Impact of type of full-field digital image on mammographic density assessment and breast cancer risk estimation: a case-control study
Source: Breast Cancer Res. 2016 Sep 26;18:96. doi: 10.1186/s13058-016-0756-7 (PMC5037867; doi:10.1186/s13058-016-0756-7)
Supplement: Additional file 6: — Quintile agreement in the percent density estimates yielded by the various density assessment method/image type combinations in control women. (DOCX 16 kb) [file 13058_2016_756_MOESM6_ESM.docx]

**Additional file 6:** Quintile agreement in the percent density estimates yielded by the various density assessment method/image type combinations in control women

| **Method** | **Type of digital image** | **Area-based methods** | | | | | | | | | |
| --- | --- | --- | --- | --- | --- | --- | --- | --- | --- | --- | --- |
|  |  | **Cumulus** | | | | | | **Libra** | | | |
|  |  | **Raw**  **(n=684)** | | **Processed**  **(n=584)** | | **Analogue-like**  **(n=684)** | | **Raw**  **(n=684)** | | **Processed**  **(n=584)** | |
|  |  | **Same Q** | **Same ± 1Q** | **Same Q** | **Same ± 1Q** | **Same Q** | **Same ± 1Q** | **Same Q** | **Same ± 1Q** | **Same Q** | **Same ± 1Q** |
| **Cumulus** | **Raw**  **(n=684)** | - | - | 62%  (361/584) | 96%  (558/584) | 57%  (392/684) | 93%  (636/684) | 45%  (308/684) | 81%  (555/684) | 48%  (283/584) | 89%  (520/584) |
|  | **Processed**  **(n=584)** | - | - | - | - | 52%  (303/584) | 93%  (544/584) | 41%  (242/584) | 83%  (482/584) | 47%  (275/584) | 87%  (510/584) |
|  | **Analogue-like**  **(n=684)** | - | - | - | - | - | - | 41%  (283/684) | 81%  (556/684) | 50%  (292/584) | 87%  (508/584) |
| **Libra** | **Raw**  **(n=684)** | - | - | - | - | - | - | - | - | 47%  (272/584) | 64%  (376/584) |
|  | **Processed**  **(n=584)** | - | - | - | - | - | - | - | - | - | - |

n: number of women; Q: quintile
