# Supplementary material for: Impact of type of full-field digital image on mammographic density assessment and breast cancer risk estimation: a case-control study
Source: Breast Cancer Res. 2016 Sep 26;18:96. doi: 10.1186/s13058-016-0756-7 (PMC5037867; doi:10.1186/s13058-016-0756-7)
Supplement: Additional file 8: — Mutually-adjusted associations of known determinants of mammographic density with absolute density readings in control women. BMI body mass index, HT hormonal therapy, OC oral contraceptives, P-value P for linear trend, No. SDs number of standard deviations (on the transformed scale). (PDF 63 kb) [file 13058_2016_756_MOESM8_ESM.pdf]

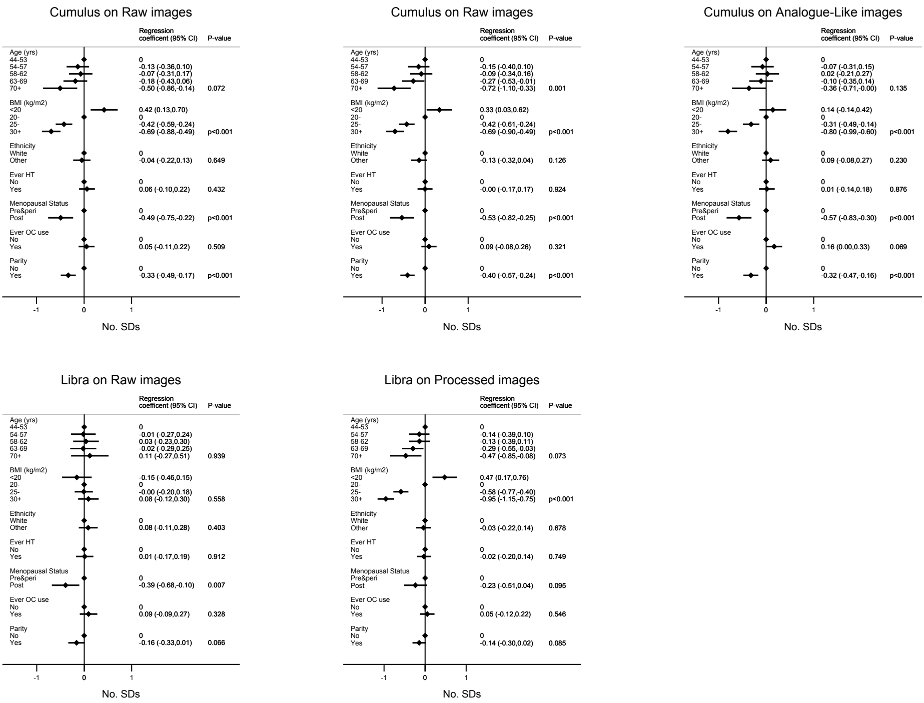
Cumulus on Raw images
Regression coefficent (95% CI)
P-value
Age (yrs)
44-53 0
54-57 -0.13 (-0.36,0.10)
58-62 -0.07 (-0.31,0.17)
63-69 -0.18 (-0.43,0.06)
70+ -0.50 (-0.86,-0.14) 0.072
BMI (kg/m2)
<20 0.42 (0.13,0.70)
20- 0
25- -0.42 (-0.59,-0.24)
30+ -0.69 (-0.88,-0.49) p<0.001
Ethnicity
White 0
Other -0.04 (-0.22,0.13) 0.649
Ever HT
No 0
Yes 0.06 (-0.10,0.22) 0.432
Menopausal Status
Pre&peri 0
Post -0.49 (-0.75,-0.22) p<0.001
Ever OC use
No 0
Yes 0.05 (-0.11,0.22) 0.509
Parity
No 0
Yes -0.33 (-0.49,-0.17) p<0.001
-1
0
1
No. SDs
Cumulus on Raw images
Regression coefficent (95% CI)
P-value
Age (yrs)
44-53 0
54-57 -0.15 (-0.40,0.10)
58-62 -0.09 (-0.34,0.16)
63-69 -0.27 (-0.53,-0.01)
70+ -0.72 (-1.10,-0.33) 0.001
BMI (kg/m2)
<20 0.33 (0.03,0.62)
20- 0
25- -0.42 (-0.61,-0.24)
30+ -0.69 (-0.90,-0.49) p<0.001
Ethnicity
White 0
Other -0.13 (-0.32,0.04) 0.126
Ever HT
No 0
Yes -0.00 (-0.17,0.17) 0.924
Menopausal Status
Pre&peri 0
Post -0.53 (-0.82,-0.25) p<0.001
Ever OC use
No 0
Yes 0.09 (-0.08,0.26) 0.321
Parity
No 0
Yes -0.40 (-0.57,-0.24) p<0.001
-1
0
1
No. SDs
Cumulus on Analogue-Like images
Regression coefficent (95% CI)
P-value
Age (yrs)
44-53 0
54-57 -0.07 (-0.31,0.15)
58-62 0.02 (-0.21,0.27)
63-69 -0.10 (-0.35,0.14)
70+ -0.36 (-0.71,-0.00) 0.135
BMI (kg/m2)
<20 0.14 (-0.14,0.42)
20- 0
25- -0.31 (-0.49,-0.14)
30+ -0.80 (-0.99,-0.60) p<0.001
Ethnicity
White 0
Other 0.09 (-0.08,0.27) 0.230
Ever HT
No 0
Yes 0.01 (-0.14,0.18) 0.876
Menopausal Status
Pre&peri 0
Post -0.57 (-0.83,-0.30) p<0.001
Ever OC use
No 0
Yes 0.16 (0.00,0.33) 0.069
Parity
No 0
Yes -0.32 (-0.47,-0.16) p<0.001
-1
0
1
No. SDs
Libra on Raw images
Regression coefficent (95% CI)
P-value
Age (yrs)
44-53 0
54-57 -0.01 (-0.27,0.24)
58-62 0.03 (-0.23,0.30)
63-69 -0.02 (-0.29,0.25)
70+ 0.11 (-0.27,0.51) 0.939
BMI (kg/m2)
<20 -0.15 (-0.46,0.15)
20- 0
25- -0.00 (-0.20,0.18)
30+ 0.08 (-0.12,0.30) 0.558
Ethnicity
White 0
Other 0.08 (-0.11,0.28) 0.403
Ever HT
No 0
Yes 0.01 (-0.17,0.19) 0.912
Menopausal Status
Pre&peri 0
Post -0.39 (-0.68,-0.10) 0.007
Ever OC use
No 0
Yes 0.09 (-0.09,0.27) 0.328
Parity
No 0
Yes -0.16 (-0.33,0.01) 0.066
-1
0
1
No. SDs
Libra on Processed images
Regression coefficent (95% CI)
P-value
Age (yrs)
44-53 0
54-57 -0.14 (-0.39,0.10)
58-62 -0.13 (-0.39,0.11)
63-69 -0.29 (-0.55,-0.03)
70+ -0.47 (-0.85,-0.08) 0.073
BMI (kg/m2)
<20 0.47 (0.17,0.76)
20- 0
25- -0.58 (-0.77,-0.40)
30+ -0.95 (-1.15,-0.75) p<0.001
Ethnicity
White 0
Other -0.03 (-0.22,0.14) 0.678
Ever HT
No 0
Yes -0.02 (-0.20,0.14) 0.749
Menopausal Status
Pre&peri 0
Post -0.23 (-0.51,0.04) 0.095
Ever OC use
No 0
Yes 0.05 (-0.12,0.22) 0.546
Parity
No 0
Yes -0.14 (-0.30,0.02) 0.085
-1
0
1
No. SDs
