# Supplementary material for: Impact of type of full-field digital image on mammographic density assessment and breast cancer risk estimation: a case-control study
Source: Breast Cancer Res. 2016 Sep 26;18:96. doi: 10.1186/s13058-016-0756-7 (PMC5037867; doi:10.1186/s13058-016-0756-7)
Supplement: Additional file 9: — Mutually-adjusted associations of known determinants of mammographic density with absolute non-density readings in control women. BMI body mass index, HT hormonal therapy, OC oral contraceptives, P-value P for linear trend, No. SDs number of standard deviations (on the square root transformed scale). (PDF 63 kb) [file 13058_2016_756_MOESM9_ESM.pdf]

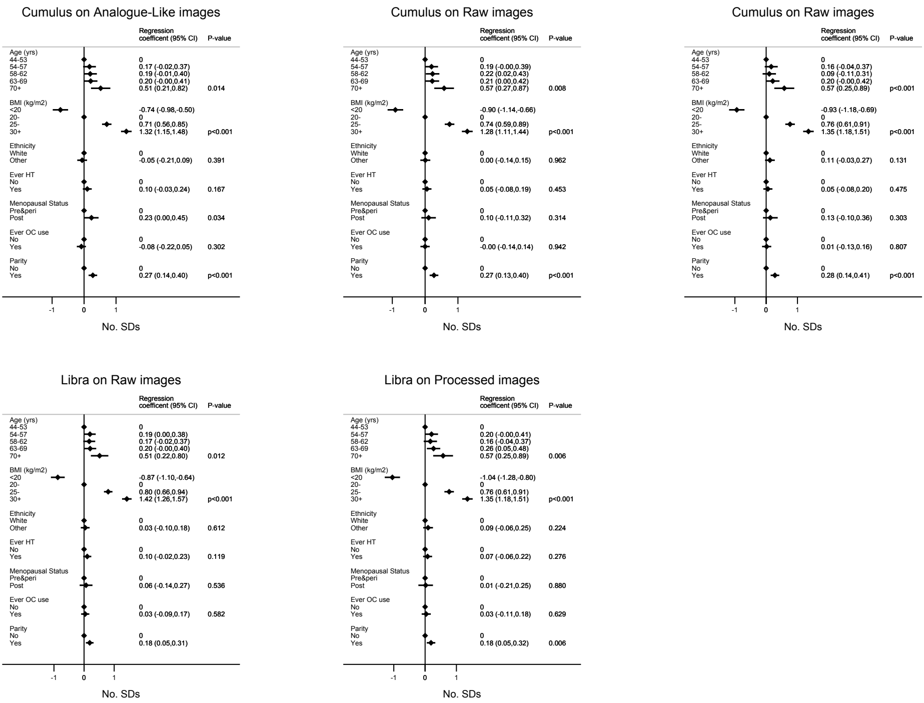
Cumulus on Analogue-Like images
Regression coefficent (95% CI)
P-value
Age (yrs)
44-53 0
54-57 0.17 (-0.02,0.37)
58-62 0.19 (-0.01,0.40)
63-69 0.20 (-0.00,0.41)
70+ 0.51 (0.21,0.82) 0.014
BMI (kg/m2)
<20 -0.74 (-0.98,-0.50)
20- 0
25- 0.71 (0.56,0.85)
30+ 1.32 (1.15,1.48) p<0.001
Ethnicity
White 0
Other -0.05 (-0.21,0.09) 0.391
Ever HT
No 0
Yes 0.10 (-0.03,0.24) 0.167
Menopausal Status
Pre&peri 0
Post 0.23 (0.00,0.45) 0.034
Ever OC use
No 0
Yes -0.08 (-0.22,0.05) 0.302
Parity
No 0
Yes 0.27 (0.14,0.40) p<0.001
-1
0
1
No. SDs
Cumulus on Raw images
Regression coefficent (95% CI)
P-value
Age (yrs)
44-53 0
54-57 0.19 (-0.00,0.39)
58-62 0.22 (0.02,0.43)
63-69 0.21 (0.00,0.42)
70+ 0.57 (0.27,0.87) 0.008
BMI (kg/m2)
<20 -0.90 (-1.14,-0.66)
20- 0
25- 0.74 (0.59,0.89)
30+ 1.28 (1.11,1.44) p<0.001
Ethnicity
White 0
Other 0.00 (-0.14,0.15) 0.962
Ever HT
No 0
Yes 0.05 (-0.08,0.19) 0.453
Menopausal Status
Pre&peri 0
Post 0.10 (-0.11,0.32) 0.314
Ever OC use
No 0
Yes -0.00 (-0.14,0.14) 0.942
Parity
No 0
Yes 0.27 (0.13,0.40) p<0.001
-1
0
1
No. SDs
Cumulus on Raw images
Regression coefficent (95% CI)
P-value
Age (yrs)
44-53 0
54-57 0.16 (-0.04,0.37)
58-62 0.09 (-0.11,0.31)
63-69 0.20 (-0.00,0.42)
70+ 0.57 (0.25,0.89) p<0.001
BMI (kg/m2)
<20 -0.93 (-1.18,-0.69)
20- 0
25- 0.76 (0.61,0.91)
30+ 1.35 (1.18,1.51) p<0.001
Ethnicity
White 0
Other 0.11 (-0.03,0.27) 0.131
Ever HT
No 0
Yes 0.05 (-0.08,0.20) 0.475
Menopausal Status
Pre&peri 0
Post 0.13 (-0.10,0.36) 0.303
Ever OC use
No 0
Yes 0.01 (-0.13,0.16) 0.807
Parity
No 0
Yes 0.28 (0.14,0.41) p<0.001
-1
0
1
No. SDs
Libra on Raw images
Regression coefficent (95% CI)
P-value
Age (yrs)
44-53 0
54-57 0.19 (0.00,0.38)
58-62 0.17 (-0.02,0.37)
63-69 0.20 (-0.00,0.40)
70+ 0.51 (0.22,0.80) 0.012
BMI (kg/m2)
<20 -0.87 (-1.10,-0.64)
20- 0
25- 0.80 (0.66,0.94)
30+ 1.42 (1.26,1.57) p<0.001
Ethnicity
White 0
Other 0.03 (-0.10,0.18) 0.612
Ever HT
No 0
Yes 0.10 (-0.02,0.23) 0.119
Menopausal Status
Pre&peri 0
Post 0.06 (-0.14,0.27) 0.536
Ever OC use
No 0
Yes 0.03 (-0.09,0.17) 0.582
Parity
No 0
Yes 0.18 (0.05,0.31)
-1
0
1
No. SDs
Libra on Processed images
Regression coefficent (95% CI)
P-value
Age (yrs)
44-53 0
54-57 0.20 (-0.00,0.41)
58-62 0.16 (-0.04,0.37)
63-69 0.26 (0.05,0.48)
70+ 0.57 (0.25,0.89) 0.006
BMI (kg/m2)
<20 -1.04 (-1.28,-0.80)
20- 0
25- 0.76 (0.61,0.91)
30+ 1.35 (1.18,1.51) p<0.001
Ethnicity
White 0
Other 0.09 (-0.06,0.25) 0.224
Ever HT
No 0
Yes 0.07 (-0.06,0.22) 0.276
Menopausal Status
Pre&peri 0
Post 0.01 (-0.21,0.25) 0.880
Ever OC use
No 0
Yes 0.03 (-0.11,0.18) 0.629
Parity
No 0
Yes 0.18 (0.05,0.32) 0.006
-1
0
1
No. SDs
