# Supplementary material for: Impact of type of full-field digital image on mammographic density assessment and breast cancer risk estimation: a case-control study
Source: Breast Cancer Res. 2016 Sep 26;18:96. doi: 10.1186/s13058-016-0756-7 (PMC5037867; doi:10.1186/s13058-016-0756-7)
Supplement: Additional file 11: — Area under the receiving operating curve (AUC) for percent and absolute density for each density assessment method/type of image combination. (DOCX 14 kb) [file 13058_2016_756_MOESM11_ESM.docx]

**Additional file 11:** Area under the receiving operating curve (AUC) for percent and absolute density for each density assessment method/type of image combination ^a^

| Method | Type of digital image | AUC ^b^ | 95% CI |
| --- | --- | --- | --- |
| *Percent density* |  |  |  |
| Cumulus | Raw | 0.62 | 0.58, 0.67 |
|  | Processed | 0.64 | 0.60, 0.69 |
|  | Analogue-like | 0.64 | 0.60, 0.69 |
| Libra | Raw | 0.61 | 0.57, 0.66 |
|  | Processed | 0.61 | 0.57, 0.66 |
| *P for difference between methods* | | 0.17 ^c^ |  |
| *Dense area* |  |  |  |
| Cumulus | Raw | 0.63 | 0.58, 0.67 |
|  | Processed | 0.65 | 0.60, 0.70 |
|  | Analogue-like | 0.65 | 0.60, 0.69 |
| Libra | Raw | 0.61 | 0.56, 0.66 |
|  | Processed | 0.62 | 0.58, 0.67 |
| *P for difference between methods* | | 0.10 ^d^ |  |

^a^ Restricted to the subset of participants of screening ages (50-69 years) and with available measurements for all five methods.

^b^ Models include age, BMI, parity and menopausal status.

^c^ Pair-wise comparisons show that the between-method differences in AUC were not statistically significant when considering measurements obtained using only Cumulus on different type of images (p=0.12) or only Libra (p=0.91).

^d^ Pair-wise comparisons show that the between-method differences in AUC were not statistically significant when considering measurements obtained using only Cumulus on different type of images (p=0.09) or only Libra (p=0.21).
